# 

**Published:** 1884-04-20

**Authors:** 


Entered at the Post-Office at Atlanta, as second-class matter.

VOL. XIV. APRIL 20, 1884. NO. 4.

THE

### SOUTHERN MEDICAL RECORD:

### A MONTHLY JOURNAL OF PRACTICAL MEDICINE.

Terms: $2.00 per Annum, in Advance; 4 Copies $6.00; Single Copies 20 Cents.

LIBRARY INDEXED S.G.O.

*"QUICQUID PRÆCIPIES ESTO BREVIS."*

### WHERE TO BUY.

| | | | |
|---|---|---|---|
| Fellows' Hypo-Phos-Phites | 1 | College for Med. Practitioners | 11 |
| Natural Uterine Supporter | 2 | Reed & Carnrick | 12 |
| Galvanic & Faradic Battery, | 3 | Harter's Iron Tonic | 13 |
| Colden's Liquid Beef Tonic | 4 | A. L. Hernstein | 14 |
| Anglo-Swiss Milk Food | 5 | Lactopeptine | 15 |
| Moore's Business University | 5 | Academic Pharmaceutic Company | 16 |
| Horlick's Food for Infants and Invalids, | 5 | Lambert & Co | (colored leaves) |
| Scott & Bowne, | 6 | J. C. Richardson | (colored leaves) |
| Warren's Food Flour | 7 | T. B. Wheeler, M. D | (colored leaves.) |
| Geo. P. Rowell & Co | 7 | J. A. McArthur | (colored leaves.) |
| Imperial Granum | 8 | A. A. Mellier | (colored leaves.) |
| French Wine Coca | 9 | Sharp & Dohme | (Inside front cover) |
| Cincinnati Sanitarium | 9 | Reed & Carnrick, | (inside back cover) |
| Battle & Co | 10 | Parke, Davis & Co | (Outside back cover) |
| University of Pennsylvania | 11 | | |

Address R. C. WORD, M.D., Managing Editor, Atlanta, Ga.

